# Investigation of Bladder Cancer Cell Response to Cryoablation and Adjunctive Cisplatin Based Cryo/Chemotherapy

**DOI:** 10.16966/2469-6714.154

**Published:** 2020-02-07

**Authors:** Kimberly L Santucci, John M Baust, Kristi K Snyder, Robert G Van Buskirk, Aaron Katz, Anthony Corcoran, John G Baust

**Affiliations:** 1 CPSI Biotech, Owego, USA; 2 Center for Translational Stem Cell and Tissue Engineering Binghamton University, USA; 3 Department of Biological Sciences, Binghamton University, USA; 4 Department of Urology, NYU Winthrop Hospital, US

## Abstract

Due to a rising annual incidence of bladder cancer, there is a growing need for development of new strategies for treatment. In 2018, the World Cancer Research Fund and other groups reported that there were ~550,000 new cases worldwide of bladder cancer. It has been further estimated that >200,000 individuals die annually from bladder cancer worldwide. Various treatment options exist. However, many if not all remain suboptimal. While the preferred chemotherapeutic options have changed in the past few years there have been few advances in the bladder cancer medical device field. Cryoablation is now being evaluated as a new option for the treatment of bladder cancer. While several studies have shown cryoablation to be promising for the treatment of bladder cancer, a lack of basic information pertaining to dosing (minimal lethal temperature) necessary to destroy bladder cancer has limited its use as a primary therapeutic option. Concerns with bladder wall perforation and other side effects have also slowed adoption.

In an effort to detail the effects of freezing on bladder cancer, two human bladder cancer cell lines, SCaBER and UMUC3, were evaluated *in vitro*. SCaBER, a basal subtype of muscle invasive bladder cancer, and UMUC3, an intermediate transitional cell carcinoma, are both difficult to treat but are reportedly responsive to most conventional treatments. SCaBER and UMUC3 cells were exposed to a range of freezing temperatures from −10 to −25°C and compared to non-frozen controls. The data show that a single 5 minute freeze to −10°C did not affect cell viability, whereas −15°C and −20°C results in a significant reduction in viability 1 day post freeze to <20%. These populations, however, were able to recover in culture. A complete loss of cell viability was found following a single freeze at −25°C. Application of a repeat (double) freeze resulted in complete cell death at −20°C. In addition to freezing alone, studies investigating the impact of adjunctive low dose (1 μM) cisplatin pre-treatment (30 minutes and 24 hours) in combination with freezing were conducted. The combination of 30 minute cisplatin pre-treatment and mild (−15°C) freezing resulted in complete cell death. This suggests that subclinical doses of cisplatin may be synergistically effective when combined with freezing.

In summary, these *in vitro* results suggest that freezing to temperatures in the range of −20 to 25°C results in a high degree of bladder cancer cell destruction. Further, the data describe a potential combinatorial chemo/cryo therapeutic strategy for the treatment of bladder cancer.

## Introduction

There is an indisputable need for the development of new strategies and medical devices for treating both local and metastatic bladder cancer. The World Cancer Research Fund and others report that there were ~550,000 new cases of bladder cancer worldwide in 2018 and it is projected that >200,000 individuals will die from bladder cancer worldwide in 2019 [1–5]. Further, bladder cancer has the highest lifetime treatment costs per patient of all cancers [6]. Although slightly less common in women, it is the fourth most common cancer in men. About half of newly diagnosed cases are non-invasive cancers contained within the inner layer of the bladder wall. About 33% of cases are more locally advanced with muscle invasion having spread into deeper layers of the bladder upon diagnosis, and the remaining cases represent metastatic bladder cancers [7]. According to the American Cancer Society the survival rate for bladder cancer varies based on the SEER stage: Localized (69%); *in situ* alone (95%); Regional (35%); Distant (5%); and 77% for all SEER stages combined [5].

Treatment for bladder cancer is dependent upon grade and stage. Low grade, early stage non-invasive cancers can be treated successfully with a combination of transurethral resection (TURBT) and intravesical therapy, where by a chemotherapeutic or immunotherapy agent is injected into the bladder for up to two hours [8]. Cancer that has grown deeply into the bladder wall or metastasized outside of the bladder is treated with radiation, systemic chemotherapy, and/or radical cystectomy. Cisplatin, 5-Fluorouracil, Mitomycin, and Gemcitabine are the most common chemotherapeutic agents used, often in some combination with or without radiation. Various treatment options exist but a number of issues have been reported, including high cost of treatment, patient discomfort, complications, incomplete treatment, procedural invasiveness, and poor efficacy, among others.

While there have been advances and shifts in the neoadjuvant chemotherapy used following radical cystectomy in patients with invasive bladder cancer (e.g. methotrexate, vinblastine, doxorubicin and cisplatin to now gemcitabine and cisplatin) [9,10], there have been few advances in ablative approaches to treat bladder cancer. Cryoablation is an established method of solid tumor ablation, offering comparable disease-free survival rates to other modalities in the treatment of prostate cancer [11] and is also used in kidney, liver, and breast cancers [12–14]. Cryoablation extracts heat from tissues and creates a dynamic thermal environment in which ultralow temperatures nearest to the cryoprobe induce cell rupture. Warmer sub-freezing isotherms extending radially from the probe’s surface result in a zone of complete necrosis, followed by a transitional zone characterized by necrotic, apoptotic and living cells due to a combination of factors including ice crystal formation, hypoxia, pH, ionic changes, energy deprivation, free radical formation, etc. [15,16]. Temperature exposure in the range of −25°C to 0°C (cancer type dependent) are characteristic of this transitional zone of heterogeneous cell death and survival. As such, this transitional zone region presents an increased probability for cancer reoccurrence *in vivo* and thereby represents a prime target for combination strategies using sensitizing agents with the goal of synergistically increasing cell death while reducing the need for prolonged freezing [16].

As a minimally invasive modality, cryoablation is an attractive option for the treatment of superficial or muscle invasive bladder cancers. A number of studies have been published showing the ability for cryoablation to freeze through the bladder wall without damage to the bladder structure [17–20]. While showing promise, the use of cryotherapy to treat bladder cancer remains limited clinically. This is due in part to a lack of data related to bladder cancer cell response to freezing, including minimal temperature and exposure time necessary (dosing) to assure lethality, concerns with perforation of the bladder wall, few cystoscopic compatible cryodevices, extended procedure time, cost and ability to target metastatic disease which limit widespread application. The purpose of this study was to investigate the freeze sensitivity of two distinct bladder cancer cell lines as an initial step in establishing dosing parameters for bladder cancer cryoablation. The SCaBER and UMUC3 cell lines were selected as SCaBER, a Squamous Cell Carcinoma (SCC), represents an aggressive basal muscle invasive bladder cancer (MIBC) whereas UMUC3, a Transitional Cell Carcinoma (TCC), represents an intermediate to high risk cancer. Analysis of the two bladder cancer variants was conducted as studies have shown that different stages and molecular dispositions of similar cancers (tissue type) can respond differently to similar treatments [21].

In addition to the potential of cryoablation, a number of studies have demonstrated the benefit of adjunctive strategies involving freezing in combination using various chemotherapy and nutraceutical agents to augment cancer cell death in various cancer types [22–25]. In the area of bladder cancer treatment, studies have shown that thermal therapy (heat and freezing) alone or in combination with chemo/radiation offers an effective approach to treat unresectable pT4b bladder cancer as well as other bladder cancer and ureteral cancer [26]. While adjunctive studies are limited, they nonetheless have shown the potential of this approach. Accordingly, in this study we also investigated the impact of the combination of low-dose (sub-clinical) cisplatin treatment in combination with mild freezing on bladder cancer cell destruction. Cisplatin was selected for combination studies as it is often used clinically to treat bladder cancer, either alone or in combination with other chemotherapeutic agents. Clinical dosage of cisplatin for the treatment of bladder cancer ranges from 35–100 mg/m^2^ [3.1–9 μM]. Given that high doses of chemotherapy cause deleterious side effects and impact patient quality of life, in this study we elected to investigate the impact of lower doses of cisplatin [1 to 1.75 μM (e.g. 11 to 19 mg/m^2^)] combined with freezing as a potential path to limit the side effects associated with drug exposure. Our hypothesis was that pre-treatment with low dose cisplatin would sensitize the bladder cancer cells so that when combined with mild freezing (−15°C) complete cell destruction would be achieved under conditions in which either the drug or freezing alone yield minimal to no impact.

This *in vitro* study was designed as a first step investigation into the sensitivity of bladder cancer to freezing as well as to evaluate the potential benefit of the combination of sub-clinical doses of cisplatin with freezing. Results detailed herein demonstrate that exposing bladder cancer cells to a single freezing event of >−25°C results in complete cell destruction with no recovery. Application of a repeat or double freeze resulted in an elevation of the minimal lethal temperature to >−20°C. Further, the combination of low dose cisplatin and a single freeze exposure resulted in an elevation of the minimal lethal temperature to −15°C.

## Materials and Methods

### Cell culture

Bladder cancer cells, SCaBER (Squamous Cell Carcinoma (SCC); ATCC HTB-3) and UMUC3 (Transitional Cell Carcinoma (TCC); ATCC CRL 1749), were cultured in T-75 flasks (Cell Treat, Shirley, MA, USA) in EMEM (ATCC 30–2003) supplemented with 10% FBS (Peak Serum, PS FB-3) and 1% penicillin/streptomycin (Lonza). Cells were lifted using TrypLE Express (Gibco/Life Technologies, Grand Island, NY), centrifuged and plated into Costar strip well plates (Corning, Tewksbury, MA, USA) at 10,000 cells per well and cultured for 2 days prior to experimentation.

### Cisplatin treatment

Cisplatin (*cis*-Diamineplatinum(II) dichloride, SigmaAldrich #479306, St. Louis, MO) was prepared fresh in sterile water prior to each use and diluted to final sub clinical concentration of 1 μM (11.6 mg/m^2^) or 1.75 μM (19.5 mg/m^2^) in media. Samples were exposed to a single application of cisplatin for 24 hours or 30 minutes prior to freezing.

### Freezing protocol

Samples in Costar 8-well strips (75 μL medium/well) were exposed to freezing temperatures of −10°C, −15°C, −20°C or −25°C in a refrigerated circulating bath (Neslab/Thermo Scientific, Waltham, MA) for 5 minutes. 30 minutes prior to freezing, culture medium was aspirated and replaced with 75 μL per well of appropriate culture medium. Strips were placed into aluminum blocks, containing a thin coating of ethanol to facilitate complete contact and thermal exchange with each well, within the baths. Ice nucleation was initiated at −2°C using liquid nitrogen vapor to prevent super cooling. Sample temperature was recorded at 1 second intervals using a type T thermocouple (Omega HH806AU, Omega, Stamford CT). For single freeze conditions, samples were held for a total time of 5 minutes in the freezing bath, passively thawed at room temperature for 10 minutes under a laminar flow hood and then placed at 37°C for recovery and assessment. For repeat (double) freeze conditions, samples were held for 5 minutes, passively thawed for 10 minutes, and then frozen again for an additional 5 minutes (5/10/5 protocol). Following the second freeze interval, samples were passively thawed at room temperature for 10 minutes and then placed into 37°C for recovery and assessment.

### Viability assessment

The metabolic activity indicator alamarBlue (Invitrogen, Carlsbad CA) was utilized to assess cell viability. Stock alamarBlue was diluted 1:20 in Hank’s Balanced Salt Solution (HBSS, Corning/Mediatech) and applied to samples for 60 min (±1 min) at 37°C. Raw fluorescent units were obtained using a TECAN Infinite plate reader (excitation 530 nm and emission 590 nm, Tecan Austria GmBH, Grodig, Austria) and analyzed using Microsoft Excel. Raw fluorescence units were converted to percentages based upon pre-freeze control values (±SEM). Assessments were conducted on day 1, 3, 5 and 7 of recovery. A minimum of 3 experimental repeats with an intra experimental repeat of 7 wells was performed in each condition (n ≥ 21). Statistical significance was determined by single factor ANOVA where p < 0.01 was applied as the significance threshold.

### Cell death assessment

Cell Event Green (ThermoFisher, C10423) and SytoxRed (ThermoFisher, S11380) were used to assess the modes (apoptosis and necrosis, respectively) and timing of cell death at 4 hours, 1, 3 and 5 days post-freeze. Cell Event Green (caspase 3/7 activation) was used to assess apoptotic involvement whereas Sytox Red indicated necrosis. Samples were incubated for 30 minutes at 37°C with 5 μM Cell Event in 1X PBS, followed by a 15 minute 37°C incubation with 500 nM Sytox Red. Samples were counterstained with 3 μg/mL Hoechst 33342 (Invitrogen, H3570) in 1X PBS and then washed for 5 minutes in 1X PBS. Samples were then analyzed with the Cell Insight CX5 high content screening platform (ThermoFisher) using the Target Activation Assay protocol. A minimum of 3 experimental repeats with an intra experimental repeat of 3 wells was performed in each condition (n ≥ 9). Statistical significance was determined by single factor ANOVA.

## Results

### Single freeze exposure response of bladder cancer cells

In order to identify the minimal lethal temperature for bladder cancer, SCaBER and UMUC3 cells were exposed to a single 5 minute freeze at −10°C, −15°C, −20°C or −25°C, thawed, allowed to recover in culture and assessed for initial cell viability (24 hours) as well as recovery over a 7 day period. Analysis of SCaBER samples revealed minimal death following exposure to a −10°C single freeze (Figure 1A). Exposure to −15°C resulted in a significant decline in SCaBER viability at day 1 post-freeze to 16% (± 1.0). The surviving cells, however, were found to rapidly recover reaching 84% (±2.2) by day 7. Exposure to −20°C resulted in a further reduction in SCaBER viability to 2% (±0.1) 1 day post freeze. A low but significant level of regrowth was observed over the 7 day post-thaw analysis interval (D7=7% (±0.8) *vs*. D1=2% (±0.2); P<0.01). When SCaBER cells were treated with a single freeze at −25°C, complete cell death at day 1 was observed and no regrowth was noted over the 7 day recovery period (D1 survival=0.2% (± 0.01), D7=0% (± 0.1), P>0.5).

Studies using the UMUC3 cell line (TCC) yielded similar results as the SCaBER studies. Exposure to a single freeze at −10°C yielded minimal cell death and −15°C resulted in day 1 survival of 22.8% (± 0.3) which increased to 97.5% (±1.1) by day 7 (Figure 1B). Exposure to −20°C resulted in a further decline in day 1 viability with a low level of recovery noted by day 7 (D1=3.2% (±0.2) *vs*. D7=17.3% (±0.7); P<0.01), whereas exposure to −25°C yielded complete cell death and no recovery. The results from these experiments suggested that for a single freeze episode the minimal lethal temperature for bladder cancer (SCC and TCC) is −25°C.

### Repeat (Double) freeze exposure response of bladder cancer cells

**Figure 2:**
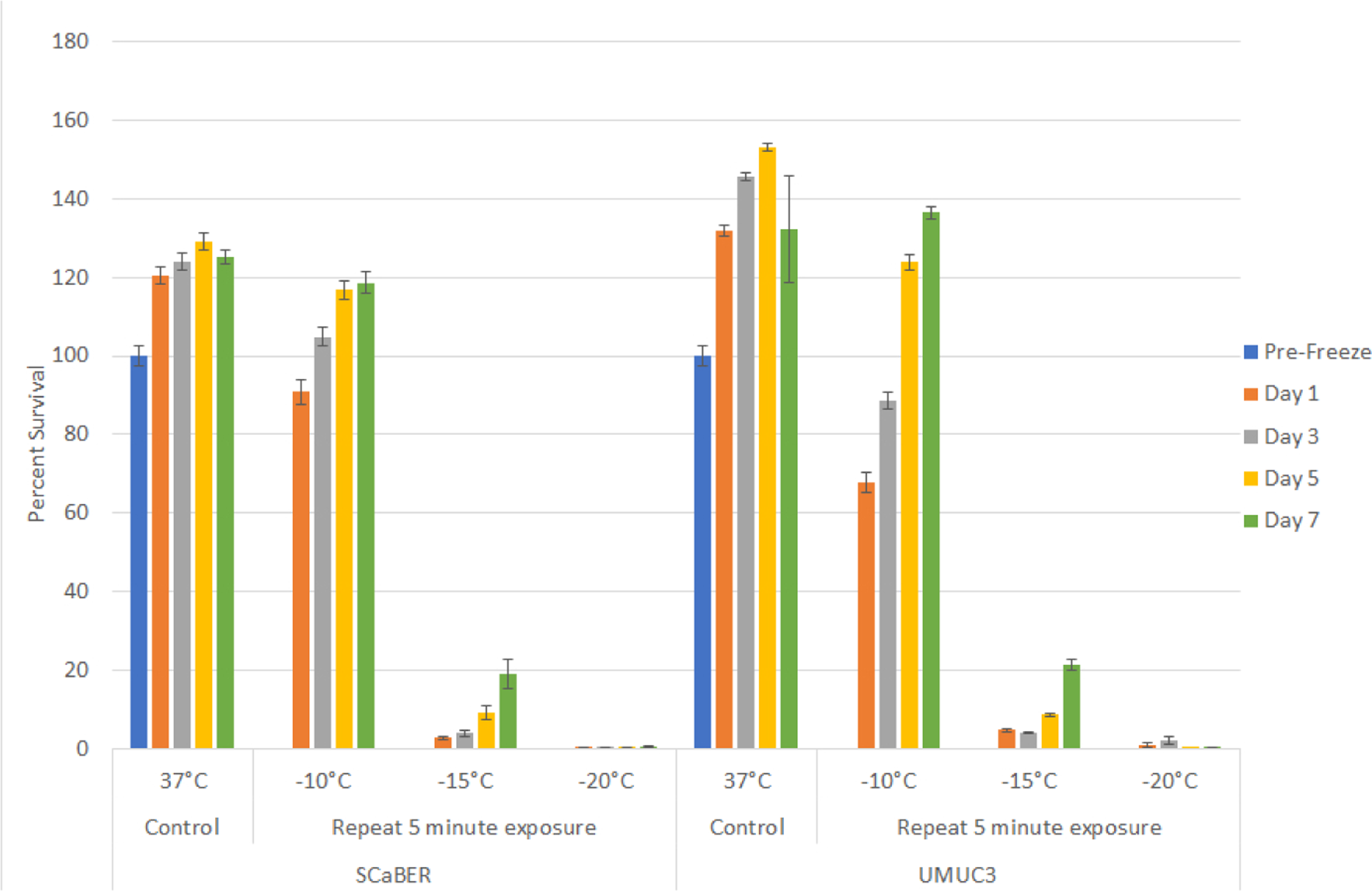
Impact of repeat freeze on bladder cancer cell viability and recovery. SCaBER and UMUC3 cells were subjected to a double 5 minute freeze (5/10/5) at −10, −15, −20, and −25°C and survival was assessed over seven days post-treatment. Data suggest that a double freeze at −20°C results in complete bladder cancer cell death with no recovery. Double freeze to −15°C resulted in a significant decrease in cell survival. However a low level of recovery was noted over the 7 day assessment interval.

With the identification of −25°C as completely lethal for SCaBER and UMUC3 cells, studies were conducted to assess the impact of a repeat (double) freeze exposure on cell viability and recovery. To this end, samples were exposed to repeat freezing at-10, −15 and −20°C. Repeat freeze exposure (double 5 minute freezes) to −10°C yielded a similar outcome as the single freeze exposure within minimal cell death observed in either the SCaBER or UMUC3 cells (Figure 2). In the UMUC3 samples, an initial decrease in viability to 67.9% (± 2.6) was noted. These samples, however, recovered to control levels by Day 5 recovery. Repeat freezing at −15°C resulted in a significant increase in cell death in both the SCaBER and UMUC3 samples at day 1 post freeze compared to a single freeze exposure (repeat *vs*. single=SCaBER: 2.8%(±0.3) *vs*. 16%(±1), P<0.01; UMUC3: 4.7%(±0.3) *vs*. 22.8%(±0.3), P<0.01). The repeat −15°C samples were found to recover to around 20% over the 7 day recovery interval (D7: SCaBER: 19.1%(±3.7); UMUC3: 21.5%(±1.3)). While slight, this recovery was significant compared to day 1 survival for both cell types (D1 *vs*. D7 P<0.01 for both cell types). Repeat exposure to −20°C resulted in complete cell destruction with no recovery over the 7 day assessment interval in both cell types. The results from the double freeze experiments suggested that the repeat exposure results in an elevation of the minimal lethal temperature to around −20°C.

### Impact of adjunctive cisplatin and freezing treatment

With the identification of the minimal lethal temperature for SCaBER and UMUC3 cell destruction and the elevation of this temperature when a repeat freeze was applied, we explored combining low dose cisplatin pretreatment followed by freezing to further increase cell death. Given the significant decrease in viability observed following exposure to −15°C with a subsequent population recovery over the 7 day interval, we chose to investigate the combination of cisplatin and freezing at −15°C. SCaBER and UMUC3 samples were exposed to low dose cisplatin for 24 hours prior to freezing. Cisplatin dosages of 1 μM for SCaBER cells and 1 μM and 1.75 μM for UMUC3 cells were examined. These dosages were selected as dose response studies revealed minimal to no negative effect on cell survival or recovery over the 7 day assessment period (data not shown). SCaBER cells exposed to 1 μM cisplatin for 24 hours followed by a single freeze at −15°C (C/−15) resulted in an increase in cell death compared to the −15°C freeze (−15) alone samples (C/−15: 10.5% (± 0.8) *vs*. −15: 16% (±1.1); P<0.01) (Figure 3A). While a significant decrease in day 1 post-freeze viability was noted, as in the −15°C freeze only condition, the cisplatin/ −15°C samples were found to recover over the 7 day interval, however to a much lower degree than in the freeze only samples (D7: C/−15: 33%(±5.4) *vs*. −15: 84%(±2.2); P<0.01)). As recovery was noted in the cisplatin ±15°C samples, experiments examining the impact of a repeat freeze at −15°C (−15R) in combination with cisplatin pretreatment (C/−15R) were conducted. These studies revealed similar day 1 survival to repeat freeze alone at −15°C (C/−15R: 4.4% (±1.4) *vs.* −15R: 2.8% (±0.3), P=0.3). Interestingly, SCaBER samples exposed to cisplatin and repeat −15°C freezing did not recover over the 7 day assessment period whereas the repeat freeze only samples did (D7: C/−15R: 3.7% (±1.2) *vs*, −15R 19.1%(±7.2); P<0.01).

Combination studies with 24 hour pretreatment using cisplatin followed by exposure to −15°C in the UMUC3 cell line yielded similar results as SCaBER samples. UMUC3 cells exposed to cisplatin (1 μM or 1.75 μM) alone for 24 hours resulted in minimal cell death (Figure 3B). When 1 μM cisplatin treatment was followed by −15°C freezing, a slight decrease in cell survival was noted at day 1 compared to freeze only samples (C/−15: 20% (±0.5) *vs* −15: 22.8% (±0.3); P=0.006)). While similar at day 1, analysis at day 7 revealed a marked decrease in sample recovery in the cisplatin/freeze combination condition *vs* freeze alone (C/−15: 62.4% (±2) *vs*. −15: 97.5% (±1.1), P<0.01). Pretreatment with 1.75 μM cisplatin for 24 hrs followed by freezing to −15°C (C1.75/−15) resulted in similar day viability as the 1 μM cisplatin/freeze and freeze alone samples (C1.75/−15: 19.4% (±1.0) *vs*. C/−15: 20 (±0.5) *vs*. −15: 22.8% (±0.3), respectively). Interestingly, the increase in cisplatin to 1.75 μM in combination with freezing resulted in a further reduction in cell recovery over the 7 day interval compared to the 1 μM cisplatin/freeze samples (D7: C1.75/−15: 34.7%(±2.4) *vs*. C/−15: 97.5% (±1.1); P<0.01).

### Impact of cisplatin exposure interval on cell survival

Based on the benefit of the combination of 24 hour exposure to low dose cisplatin prior to freezing to −15°C, we investigated the impact of shortening the cisplatin exposure interval from 24 hours to 30 minutes prior to freezing. As with 24 hour exposure, 30 minute exposure to cisplatin alone resulted in minimal cell death for both SCaBER and UMUC3 samples (Figures 3A and 3B; 30 min). The 30 minute exposure interval followed by −15°C freezing in SCaBER samples yielded similar survival to freeze alone samples (17.9% (±1.8) *vs*. 16% (±1.1), P>0.05) which represented an increase in day 1 survival compared to 24 hour pretreatment samples (17.9% (±1.8) *vs*. 10.7% (±0.8); P=0.008). Analysis over the 7 day recovery interval revealed that while the initial survival of the 30 min cisplatin exposure samples was greater than the 24 hour exposure, a decrease in sample viability was noted over the 7 day interval in the 30 min exposure/−15°C samples (D1: 17.9% (±1.8) *vs*. D7: 9.3% (±2.6); P<0.01; Figure 3A). This differed significantly from the 24 hour exposure/−15°C samples which were found to repopulate over the 7 day recovery interval. Analysis of the impact of a 30 minute cisplatin exposure followed by repeat freezing at −15°C revealed minimal cell survival and no recovery over the 7 day assessment interval. This was similar to the 24 hour cisplatin/−15°C repeat freeze condition yet differed from the −15°C alone samples.

Analysis of the 30 minute cisplatin exposure prior to −15°C freezing in UMUC3 samples revealed similar results as SCaBER samples. Exposure to 1 μM cisplatin for 30 minutes followed by freezing resulted in a similar day 1 survival as in the freeze alone and 24 hour cisplatin/−15°C samples (25.2% (±1.3) *vs*. 22.8% (±0.3) and 20.1% (±0.5), respectively) (Figure 3B). Analysis over the 7 day recovery interval revealed that, as in the SCaBER samples, UMUC3 samples exposed to cisplatin for 30 minutes and then frozen to −15°C yielded a plateau in cell survival (D7: 31.5% (±2.7) *vs*. D1: 25.2% (±1.3), P<0.01; Figure 3B). Increasing the cisplatin concentration to 1.75 μM with a 30 min exposure and then freezing resulted in a similar day 1 survival as the 1 μM samples (25.0% (±1.4) *vs*. 25.28% (±1.3), respectively). However a decline in sample viability was noted in the 1.75 μM cisplatin/−15°C samples over the 7 day assessment interval which differed from the plateau in the 1 μM combination samples. The plateau/decline in survival during the recovery interval in the 1 μM and 1.75 μM 30 minute cisplatin / −15°C freeze samples differed significantly from the 24 hour cisplatin/−15°C combination or 15°C alone samples which were both found to regrow.

### Assessment of modes of cell death following freezing

**Figure 4:**
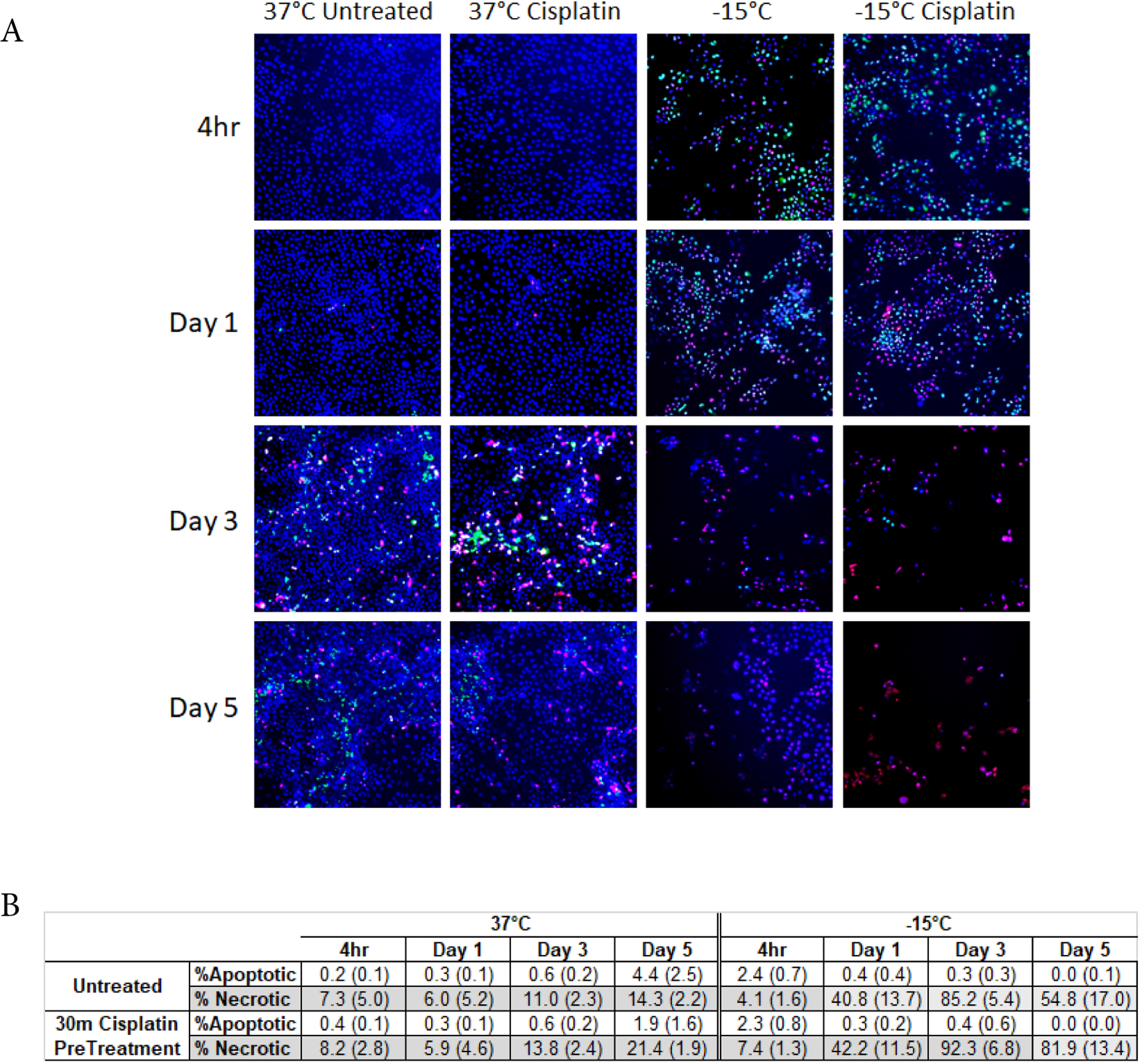
Analysis of the modes of cell death following freezing to −15°C with and without cisplatin pre-treatment. SCaBer cells were exposed to −15°C freezing for 5 mins with or without a 30 pre-treatment with Cisplatin (1μM) and then assessed for apoptotic, necrotic and living populations using CellEvent Green, SytoxRed and Hoechest, respectively (A). Quantitative image analysis revealed a significant increase in necrosis at days 1–3 post freeze in both samples. Importantly, necrotic levels were found to be higher and sustained longer in the cisplatin/−15°C samples resulting in increased cell death (B).

With the identification of increased SCaBER cell death following the combination of −15°C and cisplatin pre-treatment coupled with the observed decline in sample viability over the 5 day recovery period, analysis of the modes and timing of cell death was assessed *via* fluorescence image analysis. To this end, SCaBER samples were frozen to −15°C freeze with and without cisplatin pretreatment and analyzed at 4 hours, 1, 3 and 5 days post-freeze with Cell Event Green (apoptosis) and Sytox Red (necrosis) (Figure 4). Quantitative image analysis revealed a low level of necrosis and minimal apoptosis in both untreated controls and cisplatin control populations over the entire assessment interval (Figure 4B). Analysis of −15°C samples 4 hours post-thaw revealed minimal necrosis and apoptosis (4.1% (±1.6) and 2.4% (±0.7), respectively). Analysis at day 1 revealed a significant increase in the necrotic population to 40.8% (±13.7) compared to both 4 hour and non-treated controls (P<0.01). The necrotic population was found to continue to increase and peak at 3 days post treatment at 85% (±5.4) and then declined to 54.8% (±17.0) by day 5. No significant change in apoptotic activity was observed at any of the time points analyzed in the −15°C freeze alone samples. Analysis of cisplatin/−15°C combination samples revealed similar levels of necrosis and apoptosis as in −15°C only samples at 4 hours and 1 day post-freeze. While similar at 4 hours and day 1 post-freeze, analysis at 3 days post freeze revealed an increase in necrosis in the combination samples compared to freeze alone. Importantly, at day 5 combination samples were found to maintain a high level of necrosis (81.9% (±13.4)) whereas in the freeze only samples necrotic samples had decreased significantly (54.8% (±17.0) (P<0.01).

## Discussion and Conclusion

This study investigated the survival response of two bladder cancer cell lines following a freezing insult in an effort to identify the minimal lethal temperature (dose) necessary for complete cell destruction as well as the impact of double freeze exposure. These studies were conducted as cryoablation is often applied in a repeat (double) freeze procedure for the treatment of many cancers including prostate, renal and liver [11–14,16,27]. This dosing information could play an important guidance role for the future application of cryoablation to treat bladder cancer enabling expanded application while reducing the risk of negative side effects associated with over freezing. Further, investigation into the impact of low-dose (sub-clinical) cisplatin pre-treatment in combination with mild freezing (−15°C) were also conducted as a number of studies have detailed the benefit of adjunctive drug/freezing in enhancing cancer kill (elevating the minimal lethal temperature) under conditions which when applied as a monotherapy (freeze or drug alone) are non-lethal [16,22–25,28–38].

Investigations were conducted using two different molecular variant bladder cancer cell lines (SCaBER and UMUC3). The SCaBER cell line is derived from a squamous cell carcinoma and represents an aggressive basal Muscle Invasive Bladder Cancer (MIBC) whereas the UMUC3 cell line represents a Transitional Cell Carcinoma (TCC) which is an intermediate to high risk cancer. Previous studies have shown that different molecular variants of cancer from the same tissue can have a differential response to mild freezing thereby impacting the minimal lethal temperature [16,21,27]. For instance, in prostate cancer, studies have shown that the loss of androgen receptor expression (shift from hormone responsive to unresponsive cancer) or increase in integrin expression can result in increased tolerance to freezing shifting the minimal lethal temperature from −25°C to −40°C [21,39]. Further, studies have shown that cancer cells in general have a higher tolerance to freezing compared to non-cancerous counterparts [27,40]. As such, the SCaBER and UMUC3 cell lines were employed to determine if a difference in freeze response was observed in bladder cancer.

Initial freeze dose response studies examined bladder cancer cell survival following exposure to temperatures ranging from −10°C to −25°C. These studies revealed that both SCaBER (SCC) and UMUC3 (TCC) cells were completely destroyed following a single 5 minute freeze at −25°C whereas exposure to −10°C resulted in minimal to no cell death (Figures 1A and 1B). Single freeze exposure to the intermediate temperatures of −15°C and −20°C resulted in a significant level of cell death at 1 day post-freeze. However, both cell types were found to recover from this treatment over the 7 days post treatment assessment interval. As cryoablation is often applied using a double freeze protocol clinically, freeze response studies using a repeat freeze protocol, double 5 min freeze with an intermediate 10 min thaw (5/10/5), were conducted. These studies revealed that the application of a double freeze resulted in an increase in cell death and a reduction in sample repopulation following exposure to −15°C (Figure 2). Importantly, the application of a double freeze protocol resulted in complete cell death at −20°C (elevation of the minimal lethal temperature). This 5°C elevation in the minimal lethal temperature is significant as when correlated with the published isothermal distribution within a typical ice ball produced by a argon-based JT cryosystem represents a shift in the destructive volume from ~36% of the frozen mass to ~48%, a ~25% increase in the destructive volume [41–43].

Numerous studies have detailed the benefit of adjunctive treatment involving cryoablation with the pre-treatment of low-dose chemotherapy, nutraceuticals or other agents in a number of cancers including prostate, breast, lung and liver among others [24,25,28–36]. *In vitro* and *in vivo* studies involving prostate cancer have shown the ability to elevate the minimal lethal temperature for hormone refractory prostate cancer from −40°C to −20°C *via* pre-treatment with sub-clinical (non-toxic) doses of 5-fluorocil, taxotere and Cisplatin [24,28,33]. Other studies have demonstrated that combinatorial approaches using the active nutraceutical Calcitriol (Vitamin D_3_) can result in the elevation of the minimal lethal temperature for prostate cancer to the −10 to −15°C range [22, 23,37,38]. Based on these reports and the current usage of cisplatin as a primary treatment for bladder cancer, we investigated the potential of combining low dose cisplatin pre-treatment with mild freezing.

Cisplatin elicits its antitumor effects by damaging DNA through intra- and inter-strand cross links. These DNA adducts interfere with DNA replication and transcription, activating various DNA repair mechanisms within the cell. When repair cannot be executed the cellular stress induced by DNA damage activates the intrinsic mitochondrial mediated apoptotic pathway [44]. Freezing cells to temperatures reflective of the periphery of a cryogenic lesion, −10°C to −25°C, has also been shown to induce the intrinsic apoptotic pathway [45,46]. As such, we hypothesized that the combination of freezing and cisplatin would amplify cell death signaling resulting in increased destruction. Given that chemotherapy is typically administered locally to the bladder when feasible, the combination of low dose cisplatin and cryoablation could be a powerful strategy to improve outcomes, even in patients with more aggressive cancer types. Exposure times of 30 mins and 24 hours prior to freezing were selected as these represent clinically relevant, convenient, short exposure times to further reduce the exposure interval. In this study we investigated cisplatin doses of 11 and 19 mg/m^2^ (1 and 1.75 μM) which represents ~1/3 to 1/8 the typical clinical dosage range (35–100 mg/m^2^) for bladder cancer [8]. Subclinical doses were studied in an effort to increase cancer susceptibility to freezing injury while reducing or eliminating the negative toxic side effects associated with chemotherapy.

Pretreatment of SCaBER and UMUC3 populations with cisplatin 24 hours prior to freezing to −15°C resulted in a decrease in cell survival at Day 1 post-freeze compared to freeze alone. Yet these populations began to repopulate over the 7 day recovery interval (Figures 3A and 3B). The combination of 24 hour pre-treatment and a double freeze at −15°C resulted in complete cell destruction (Figure 3A), shortening the exposure time to 30 minutes prior to freezing at −15°C resulted in a shift in SCaBER and UMUC3 cell response from initial death followed by recovery, as observed in the −15°C alone and 24 hour cisplatin/−15°C samples, to a high degree of initial death and no repopulation (Figures 3A and 3B). Interestingly, in the case of the 30 minute 1 μM cisplatin/−15°C SCaBER and 1.75 μM/−15°C UMUC3 samples, a continued decline in viability was observed over the 7 days recovery interval. This differed from any of the other conditions where either minimal death (cisplatin alone) or initial death followed by recovery (−15°C, 24 hr. cisplatin/−15°C) was observed. Fluorescence image analysis of −15°C and cisplatin/−15°C samples revealed that the combination treatment resulted in a significant increase in necrotic activity, which was sustained over the entire 5 day assessment period (Figure 4A). Importantly, analysis at day 5 revealed a 50% increase in necrotic activity in the cisplatin/−15°C samples over the −15°C freeze only samples (Figure 4B). We hypothesize that the application of cisplatin immediately (30 mins) prior to freezing likely prevented the initiation of cellular repair mechanisms necessary for survival, resulting in the observed delayed onset cell death whereas 24 hour cisplatin treatment prior to freezing allowed enough time for the execution of signaling pathways necessary for DNA repair, and as such the synergistic effect is lost. This shift in the minimal lethal temperature to −15°C through the combination of 30 minute cisplatin pretreatment followed by freezing at −15°C is significant, as when correlated with the published isothermal distribution within a typical iceball produced by a argon-based JT cryosystem, represents a shift in the destructive volume from ~36% of the frozen mass (−25°C isotherm) to ~58%, a ~59% increase in the destructive volume [41–43,47].

In conclusion, our findings suggest that the minimal lethal temperature for bladder cancer is −25°C for a single freeze event. Application of a repeat freeze protocol results in an increase of the minimal lethal temperature to −20°C. These temperatures were found to be lethal to both squamous cell (SCaBER) and transitional cell (UMUC3) carcinoma cells *in vitro*. Pretreatment with low dose cisplatin in combination with freezing resulted in an increase in the level of cell death and inhibition of repopulation of surviving cells. Importantly, the combination of low dose cisplatin pre-treatment for 30 minutes followed by freezing resulted in a high degree of destruction at −15°C. The data suggest that the combination resulted in a shift of the minimum lethal temperature for bladder cancer from −25°C to the −15°C range. Extrapolating these *in vitro* findings to an *in vivo* scenario, the data suggest that both freezing alone and in combination with cisplatin may provide benefit in the treatment of bladder cancer. This in turn has the potential to improve outcome while reducing co-morbidities associated with freezing (bladder wall perforation, positive freeze margins) and chemotherapy (nausea, fatigue, increased susceptibility to infection, etc.) while providing for an effective minimally invasive treatment strategy for bladder cancer. In combination with previous *in vitro* and *in vivo* reports, these data suggest cryoablation alone or in combination with low dose chemotherapy may provide an improved path for the treatment of bladder cancer.

## Figures and Tables

**Figure 1A:**
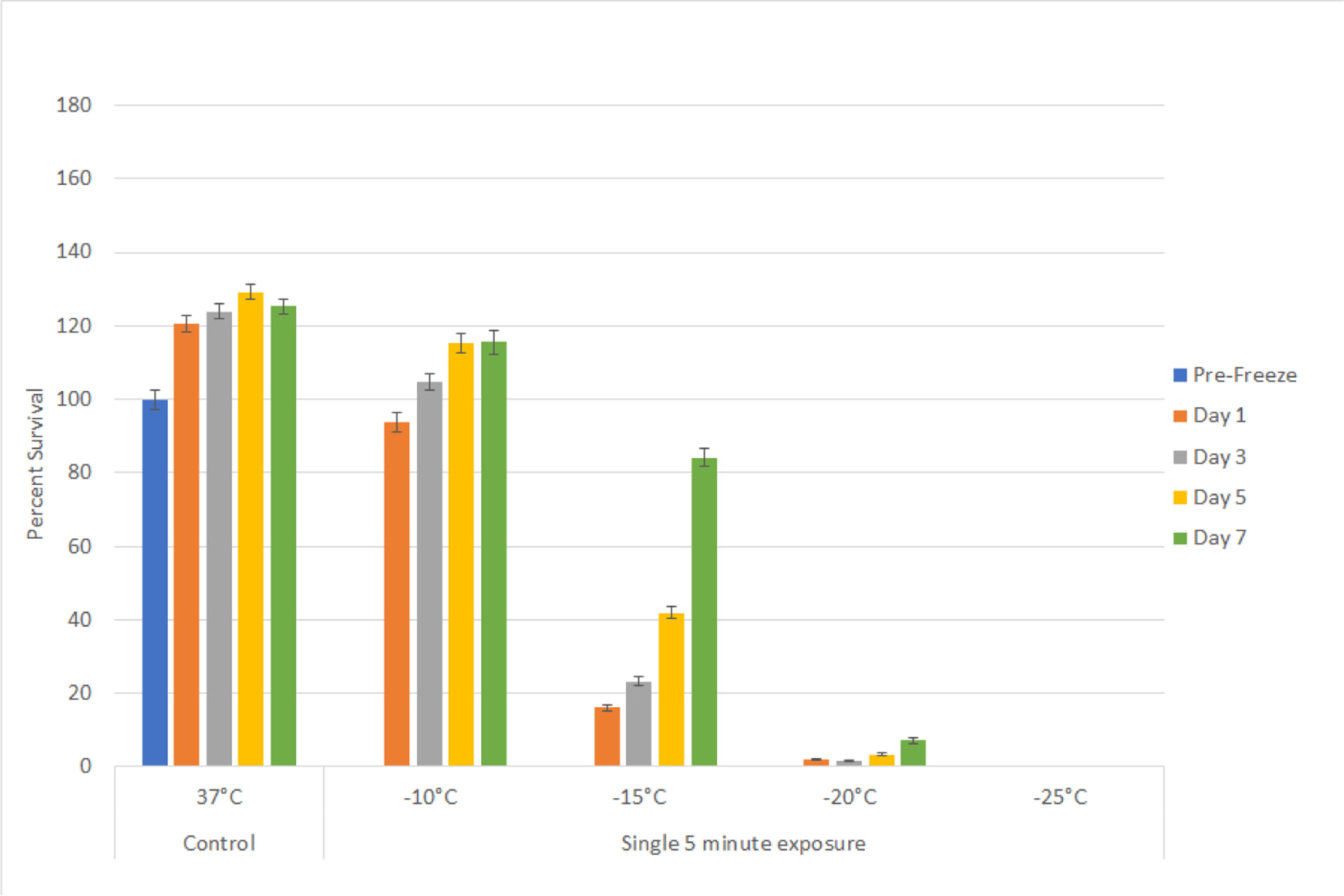
Assessment of bladder cancer cell viability and recovery following a single freeze event. SCaBER (A) cells were subjected to a 5 minute freeze at −10, −15, −20, and −25°C and survival was assessed over seven days post-treatment. Data suggest that complete cell death with no recovery is attained following exposure to −25°C whereas −15°C and −20°C exposure results in a substantial level of cell death followed by recovery in culture.

**Figure 1B:**
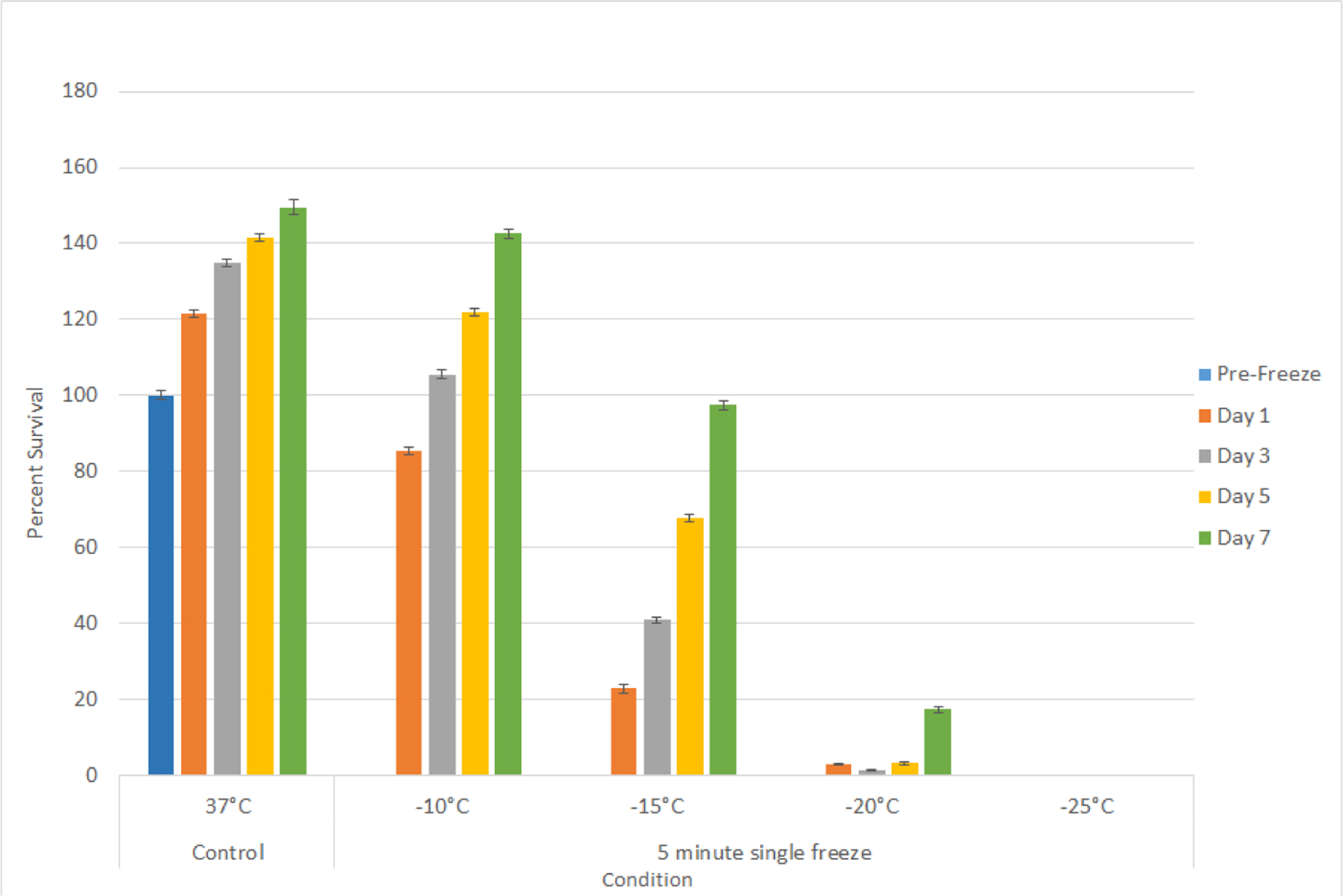
Assessment of bladder cancer cell viability and recovery following a single freeze event. UMUC3 (B) cells were subjected to a 5 minute freeze at −10, −15, −20, and −25°C and survival was assessed over seven days post-treatment. Data suggest that complete cell death with no recovery is attained following exposure to −25°C whereas −15°C and −20°C exposure results in a substantial level of cell death followed by recovery in culture.

**Figure 3A:**
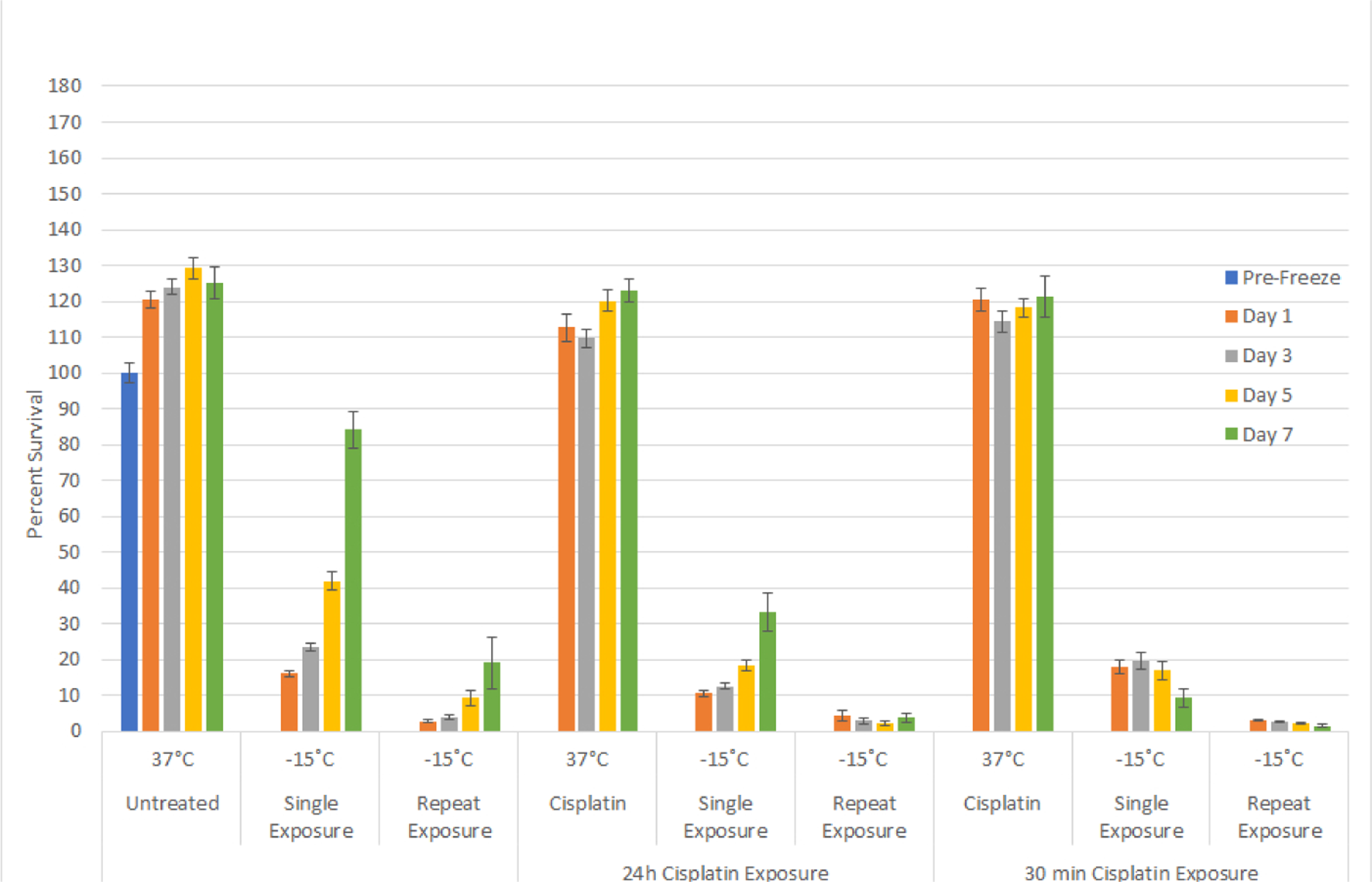
Effect of adjunctive low dose cisplatin pretreatment in combination with freezing on bladder cancer cell survival. SCaBER (A) and UMUC3 (B) cells were subjected to 30 minutes of 24 hours pretreatment with a sub-clinical dose (1μM or 1.75μM) cisplatin followed by a 5 minute freeze at −15°C. Cell survival and recovery was assessed over seven days post-treatment. Data suggest that the combination of a cisplatin 30 minute pretreatment and −15°C freezing results in complete bladder cancer cell death. While not as effect as a 30 minute pretreatment, a 24 hour cisplatin pretreatment revealed a decline in cell recovery post-freeze compared to freeze alone samples.

**Figure 3B:**
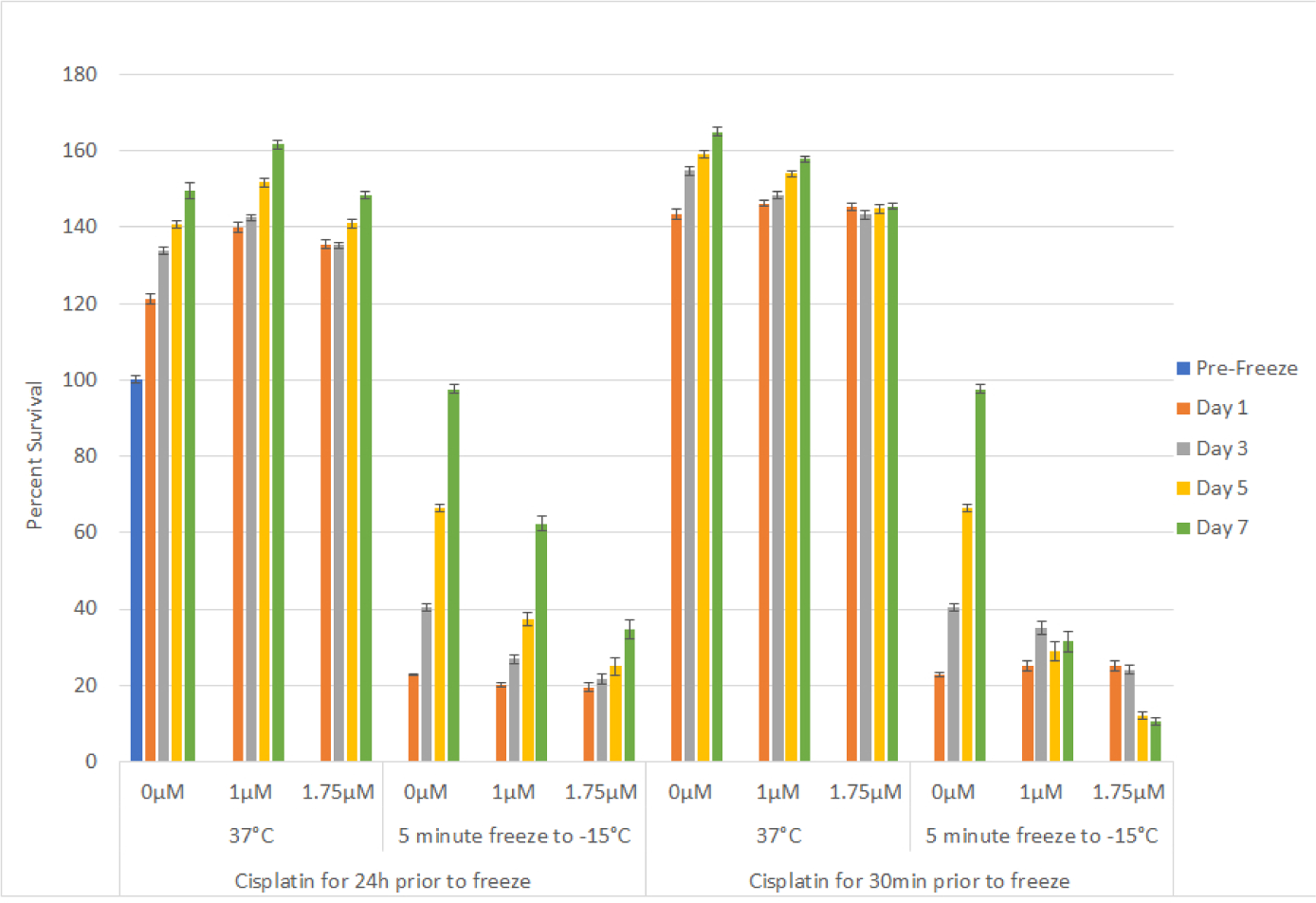
Effect of adjunctive low dose cisplatin pretreatment in combination with freezing on bladder cancer cell survival. SCaBER (A) and UMUC3 (B) cells were subjected to 30 minutes of 24 hours pretreatment with a sub-clinical dose (1μM or 1.75μM) cisplatin followed by a 5 minute freeze at −15°C. Cell survival and recovery was assessed over seven days post-treatment. Data suggest that the combination of a cisplatin 30 minute pretreatment and −15°C freezing results in complete bladder cancer cell death. While not as effect as a 30 minute pretreatment, a 24 hour cisplatin pretreatment revealed a decline in cell recovery post-freeze compared to freeze alone samples.
